# A Rare Cause of Congenital Adrenal Hyperplasia: Clinical and Genetic Findings and Follow-up Characteristics of Six Patients with 17-Hydroxylase Deficiency Including Two Novel Mutations

**DOI:** 10.4274/jcrpe.0032

**Published:** 2018-07-31

**Authors:** Aslı Derya Kardelen, Güven Toksoy, Firdevs Baş, Zehra Yavaş Abalı, Genco Gençay, Şükran Poyrazoğlu, Rüveyde Bundak, Umut Altunoğlu, Şahin Avcı, Adam Najaflı, Oya Uyguner, Birsen Karaman, Seher Başaran, Feyza Darendeliler

**Affiliations:** 1 İstanbul University İstanbul Faculty of Medicine, Department of Pediatrics, Pediatric Endocrinology Unit, İstanbul, Turkey; 2 İstanbul University İstanbul Faculty of Medicine, Department of Medical Genetics, İstanbul, Turkey; 3 İstanbul University İstanbul Faculty of Medicine, Department of Pediatrics, İstanbul, Turkey

**Keywords:** Congenital adrenal hyperplasia, CYP17A1, disorder of sex development, hypertension, primary amenorrhea

## Abstract

**Objective:**

17α-hydroxylase/17,20 lyase deficiency (17OHD) is a rare form of congenital adrenal hyperplasia (CAH), characterized by hypertension and varying degrees of ambiguous genitalia and delayed puberty. The disease is associated with bi-allelic mutations in the *CYP17A1* gene located on chromosome 10q24.3. We aimed to present clinical and genetic findings and follow-up and treatment outcomes of 17OHD patients.

**Methods:**

We evaluated six patients with 17OHD from five families at presentation and at follow up. Standard deviation score of all auxological measurements was calculated according to national data and karyotype status.* CYP17A1* gene sequence alterations were investigated in all patients.

**Results:**

The mean (±standard deviation) age of patients at presentation and follow-up time was 14.6±4.2 and 5.0±2.7 years respectively. Five patients were referred to us because of delayed puberty and primary amenorrhea and four for hypertension. One novel single nucleotide insertion leading to frame shift and another novel variant occurring at an ultra rare position, leading to a missense change, are reported, both of which caused 17OHD deficiency. Steroid replacement was started. The three patients with 46,XY karyotype who were raised as females underwent gonadectomy. Osteoporosis was detected in five patients. Four patients needed antihypertensive treatment. Improvement in osteoporosis was noted with gonadal steroid replacement and supportive therapy.

**Conclusion:**

17OHD, a rare cause of CAH, should be kept in mind in patients with pubertal delay and/or hypertension. Patients with 46,XY who are raised as females require gonadectomy. Due to late diagnosis, psychological problems, gender selection, hypertension and osteoporosis are important health problems affecting a high proportion of these patients.

## What is already known on this topic?

17-hydroxylase deficiency is a rare cause of congenital adrenal hyperplasia that presents with hypergonadotropic hypogonadism, primary amenorrhea, hypertension and hypokalemia. Data on long term follow-up of patients with 17-hydroxylase deficiency is scarce. Whether genotype and phenotype are correlated is unclear.

## What this study adds?

This study provides information about long term follow-up of patients with 17-hydroxylase deficiency and their therapeutic outcomes. We describe two novel mutations which both abolish CYP17A1 protein expression. Neither a significant similarity nor a significant difference was found between the genotype and phenotype of the study group.

## Introduction

17-hydroxylase deficiency (17OHD) is a rare form of congenital adrenal hyperplasia (CAH). It has an estimated incidence of 1 in 50 000 newborns and accounts for 1% of all CAH cases. It is the second most common form of CAH in Brazil (1), Japan (2) and China (3,4). The disease is caused by biallelic mutations in the *CYP17A1* gene, encoding the enzyme 17α-hydroxyprogesterone (OHP) aldolase (Cytochrome P450c17) catalyzing the conversion of pregnenolone and progesterone to their 17-alpha-hydroxylated products and subsequently to dehydroepiandrosterone (DHEA) and androstenedione (Δ4A) via 17α-hydroxylation and the 17,20-lyase reaction. It is the key enzyme for cortisol synthesis that is essential for sex steroid production. Absence of enzyme activity drives overproduction of pregnenolone, progesterone, 11-deoxycorticosterone (DOC) and corticosterone that leads to the mineralocorticoid effect, resulting in hypertension and hypokalemia. Excess DOC with low renin concentration causes hypertension and the lack of androgens and estrogens cause sexual infantilism and pubertal failure. The absence of 17,20 lyase activity in the adrenal gland also results in deficiency of DHEA sulfate (DHEA-S), causing failure of adrenarche and pubic and axillary hair development (5,6). Although 46,XY patients have impaired steroid synthesis, they have normal Sertoli cell function producing anti-Mullerian hormone and causing regression of Mullerian structures (7).

Complete deficiency typically causes female external genitalia, delayed puberty, hypergonadotrophic hypogonadism, primary amenorrhea, hypertension, hypokalemia and absence of pubic hair. Differential diagnosis must include cytochrome P450 oxidoreductase deficiency and androgen insensitivity syndrome in complete deficiency (5).

The *CYP17A1* gene is located on chromosome 10q24.3, reference transcript (NM_000102) and contains eight exons with a length of 1870 bp, translating into a 508 amino acid polypeptide (NP_000093) containing a cytochrome p450 domain encoded between codons 28 to 493. The disease is inherited in an autosomal recessive manner and hence parental consanguinity becomes an important risk factor (8).

Currently over 100 mutations in the *CYP17A1* gene have been identified (including point mutations, small deletions/insertions, duplications, frame shift mutations and, rarely, large deletions). Founder effects may also contribute to the high prevalence of the disease in some countries. In Brazil c.1084C>T (p.R362C) and c.1216T>C (p.W406R) mutations had the founder effect (1) while in China nonsense mutation, c.987C>A (p.Y329*), and in frame deletion of p.(D487_F489) due to c.1459_1467delGACTCTTTC, affected more than 80% of the patients (4). In spite of this founder effect, phenotype and genotype correlation is still unknown and a remarkable variation in the severity of the disorder was noted even with the same mutation (8). Interestingly, large gene deletion, namely complete exon 1-6 deletion has been identified in two families from South East Turkey which suggests that this mutation may have a founder effect for Turkey (9,10).

We report the genotypes and phenotypes of six Turkish patients with 17OHD. We aimed to identify the clinical manifestations, genetic findings and follow-up and treatment results of 17-OHD patients.

## Methods

Six Turkish patients, from five families, were included in the study. We followed these patients from 1999 to 2016 in the Pediatric Endocrinology Unit of İstanbul Faculty of Medicine. In most patients, clinical diagnosis was suspected because of the absence of secondary sex characteristics, mostly associated with the presence of hypertension and hypokalemia. The diagnosis of complete 17-OHD was established based on typical laboratory findings of reduced cortisol, plasma renin activity (PRA) and aldosterone and elevated gonadotropins and progesterone with almost absent sex steroids. All patients had been genotyped and specific CYP17A1 mutations were identified. The data, collected retrospectively, consisted of physical examination, auxological findings, hormone assays, biochemical and radiological findings, additional features at follow-up and surgical and medical treatment. Height and weight measurements of the patients were taken by the same auxologist. The parental heights were measured and an estimated target height, in keeping with the karyotype was calculated. Body mass index was calculated. At presentation for evaluation of hypertension, ambulatory blood pressure measurement was performed in all patients. At follow-up blood pressure was evaluated by a manual sphygmomanometer in the seated position or digital measurements at home. Hypertension in children and adolescents was defined as systolic and/or diastolic blood pressure, that is, on repeated measurement, ≥95^th^ percentile (11). All patients were evaluated for peripheral effects of hypertension including fundus evaluation, echocardiographic evaluation and microalbuminuria. Bone age was evaluated using the Greulich-Pyle method according to karyotype. Predicted adult height was calculated according to Bayley Pinneau method. Standard deviation score (SDS) of all auxological measurements were calculated according to national data (12) and karyotype status.

The blood samples were collected in the morning after eight hours of fasting. Hormonal evaluation included baseline 17OHP, progesterone, cortisol, Δ4A, DHEA-S, adrenocorticotropic hormone (ACTH), luteinizing hormone (LH), follicle-stimulating hormone (FSH), estradiol (E2), testosterone, PRA, aldosterone levels. Δ4A, and 17OHP levels were analyzed by radioimmnoassay. ACTH, PRA, aldosterone, LH, FSH, E2, progesterone, cortisol, DHEA-S were measured using the IMMULITE 2000 system (Siemens AG, Berlin and Munich, Germany), immunochemiluminescence assay; ICMA, Siemens. Baseline and stimulated progesterone and cortisol levels with ACTH (0.25 mg, IV Synacthen, Ciba-Geigy, Basel, Switzerland), were measured to diagnose the adrenal enzyme defect. 

Pelvic ultrasonography (USG) was performed by a pediatric radiologist using a SonoSite Titan ultrasound machine (Bothell, WA, USA) with a 5 MHz probe. 

Bone mineral density (BMD) was evaluated using dual-energy X-ray absorptiometry (DXA) (Hologic QDR 4500A Fan Beam X-ray Bone Densitometer, Hologic, Bedford, MA, USA) and analyzed using software version 12.3. BMD was measured in the spine (L1-L4). Volumetric measurements were done according to national data (13). Lumbar spine BMD z-scores between -1.0 and -2.0 define osteopenia and less than -2.0 define osteoporosis (14).

High resolution banding technique was used for karyotyping in the blood lymphocytes of all patients. Twenty metaphases were analyzed for each patient. DNA samples isolated from venous blood were investigated for disease causing mutations in all of the eight exons and exon-intron boundaries of the *CYP17A1* gene by Sanger sequencing (ABI 3500). Pathogenicity of the novel nonsynonymous mutation was analysed via *in silico* prediction programs (MutationTaster, Polyphen, SIFT). qPCR was performed in one patient because of failure to obtain PCR product for exon 1-6 of the *CYP17A1 *gene. Primer pairs for inhouse validated control gene (*CENPJ *exon 3, 6, 12) and *CYP17A1* gene (exon 1, 6 and 7) were designed to produce a minimum of 150 bp and maximum of 250 bp products with identical melting point temperature. Performance of the designed primers were first tested by cold PCR, before the qPCR reaction with EvaGreen fluorescent dye was operated in three parallel runs concurrently for index, parental and control samples for each region on CFX96 Thermal Cylcler (Bio-Rad Laboratories, Inc., CA, USA). Double delta Ct method was used for the analysis of the quantification.

Written informed consent was obtained from all patients. The study protocol was approved by the Clinical Research Ethics Committe of İstanbul University (approval number: 2016/728).

### Statistical Analysis

Statistical analysis was performed by using the SPSS 22 (IBM, Chicago, ILL, USA).

## Results

### Clinical Findings

All patients presented as phenotypical females and all had complete enzyme deficiency. Parents of Patient 4 had second degree, while others had first degree consanguineous marriage. Patients were referred to our clinic at a mean ± standard deviation (SD) (range) age of 14.6±4.2 (6.2-17.8) years. Except for one, all of them were in adolescence. Mean ± SD (range) follow-up period was 5.0±2.7 (2.2-8.9) years. Five patients presented with lack of pubertal development and primary amenorrhea (83.3%) and four patients with hypertension (66.7%). Three patients (50%) had short stature and Patient 1 and Patient 3 had severe short stature. Bone age was retarded in all patients. All patients were prepubertal, only Patient 5 was Tanner 2, because of previous use of estrogen. All patients, regardless of chromosomal sex, were raised as female. Three patients were 46,XX and three were 46,XY. 46,XY patients had a female appearance with palpable testes. The clinical findings of 17OHD patients at presentation are summarized in Table 1. 

LH and FSH levels were significantly elevated, whereas T or E2 production were blunted. Patients’ baseline progesterone and ACTH levels were high while cortisol, 17OHP and PRA were supressed. The DOC levels of three patients were measured and all were above the normal ranges. Three patients had hypokalemia during presentation (Table 1). All patients had pelvic and gonadal USG and the 46,XY patients lacked Mullerian structures. Response to ACTH stimulation test were consistent with the diagnosis of 17OHD (Table 1).

During follow-up hydrocortisone replacement therapy was started. Three 46,XY patients raised as females underwent gonadectomy. Mean (±SD) age at gonadectomy was 13.5±5.7 years. Transdermal E2 replacement was started to induce puberty in all patients. Subsequently, oral contraceptives were given to 46,XX patients to achieve regular menstrual cycles. Breast development reached stage 4-5 at 18.7±1.8 age (duration of estrogen replacement: 2.2±1.4 years). Menarche occurred at age 17.6±0.7 years in 46,XX patients. DHEAS and Δ4A levels were low in all patients and two of them did not have adrenarche (Patient 1 and Patient 2), although two patients (Patient 3 and Patient 6) had sparse pubic hair and two patients (Patient 4 and Patient 5) had pubic hair consistent with Tanner stage 3-4. 

Four patients had hypertension at presentation and were started on antihypertensive treatment (calcium channel blocker and aldosterone antagonist) (Table 2). In all patients, continuation of antihypertensive pharmacotherapy was needed, even after adrenal precursor levels decreased on hydrocortisone replacement. Echocardiography for hypertensive cardiomyopathy was normal in all patients and only Patient 2 had ASD at cardiac evaluation. Three patients had grade 1 hypertensive retinopathy (Patients 3, 4 and 6) and none of them had microalbuminuria. Antihypertensive doses were adjusted according to blood pressure monitoring. Despite attempts to wean the patients off antihypertensive therapy, this was not possible and therapy was continued.

BMD measurement revealed osteoporosis in five patients. In addition to sex steroid replacement, vitamin D and calcium treatment were initiated. Only Patient 4 needed bisphosphonate treatment, due to severe osteoporosis, and alendronate was given between the ages of 17 and 21 years. Improvement in osteoporosis was noted with sex steroid replacement and supportive therapy.

At final evaluation four patients had reached final height. Only Patient 3 with a 46,XY karyotype had short stature with a height SDS of -2.8 (final height 163.5 cm). Two patients who did not reach final height also had short stature (Patient 2 and Patient 6). Height SDS of Patient 6 was -2.0, while that of Patient 2 was -0.3. Patient 2 had short stature according to the difference between height SDS and target height SDS which was equal to -1.3. Currently these two patients are continuing to increase in height.

### Genetic Findings

**Figure 1:**
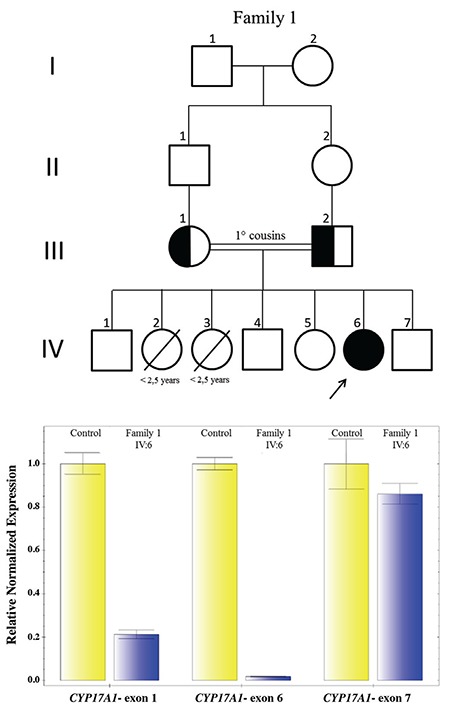
Pedigree of Family 1 and *CYP17A1* gene exon 1-6 deletion

**Figure 2:**
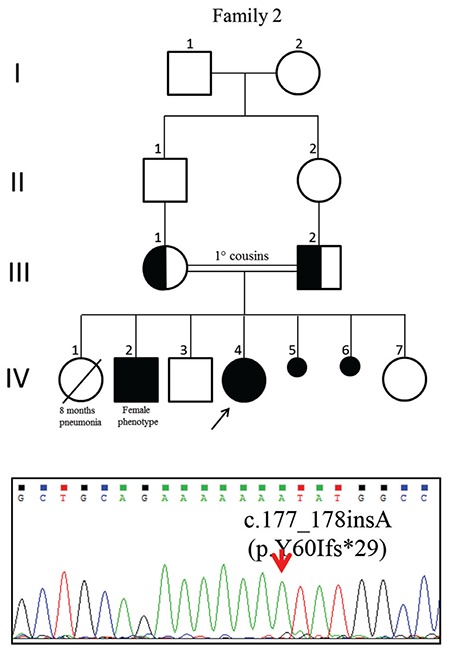
Pedigree of Family 2 and sequencing electropherograms of the *CYP17A1* gene. The upper lane shows the mutation of c.177_178insA (p.Y60Ifs*29)

**Figure 3:**
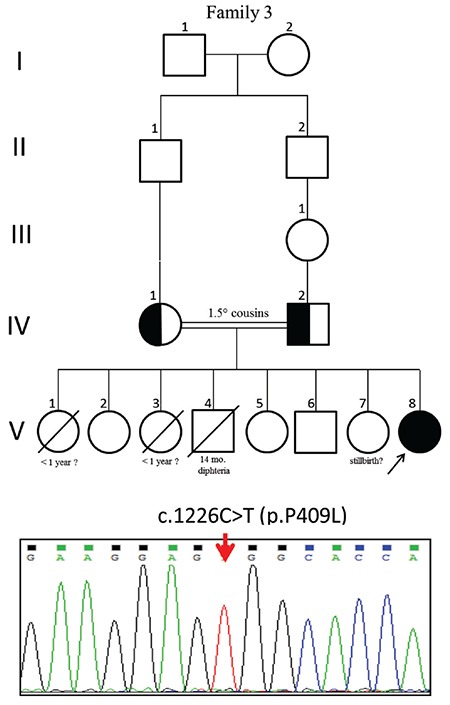
Pedigree of Family 3 and sequencing electropherograms of the *CYP17A1* gene. The upper lane shows the mutation of c.1226C>T(p.P409L)

Molecular analysis revealed five different homozygous mutations, one of which is novel and was found in two patients from the same family, c.177_178insA (p.Y60Ifs*29). Another very rare alteration, 1 in 246.050 allele, c.1226C>T (p.P409L, rs367833709) was found in one further patient (see Figures 1, 2 and 3). Parental testing showed heterozygosity for the alterations, supporting autosomal recessive inheritance. *In silico* analyses findings predicted that the c.1226C>T alteration would be disease causing (Table 1) (Figure 3).

## Discussion

In this study we report the clinical and genetic findings and follow-up characteristics of 17OHD patients from a single center. Six patients from five families, all born to consanguineous parents, presented at adolescent ages, mostly with symptoms of pubertal delay and hypertension. 17OHD, a rare form of CAH, usually presents in adolescence (8). In this study, four patients (66.7%) had hypertension and three had hypokalemia (50%) while others had not developed the symptoms of mineralocorticoid excess at presentation. Patients who suffer from this condition may develop hypertension and hypokalemia at any age, which makes the diagnosis difficult.

All six patients reported here, who had absent sex steroid activity and pubertal development, presented with complete deficiency. All patients had hypergonadotropic hypogonadism. However the defect may be partial or complete. Partial 17OHD patients have some degree of estrogenic and androgenic function. In partial forms patients have normal 17α-hydroxylase activity, while 17,20 lyase activity is absent. Patients may have spontaneous breast and pubic hair development, oligomenorrhea or secondary amenorrhea while complete forms have absent pubarche and adrenarche. Furthermore, patients with partial forms may be normokalemic and normotensive (15). 

At presentation four of our patients were hypertensive and three patients had hypokalemia. Hydrocortisone replacement at physiologic doses (8-10 mg/m^2^/day) is required for treatment. Hydrocortisone supresses precursor concentrations and improves symptoms. The dose is titrated to normalise blood pressure and potassium levels. In this cohort, following hydrocortisone treatment, hypokalemia resolved while hypertensive patients needed antihypertensive medication in addition to hydrocortisone to achieve blood pressure control. Calcium channel blockers or spironolactone may be required for hypertension refractory to hydrocortisone. Reports suggest that 10-15% of 17OHD patients may be normotensive (16). Some patients have been diagnosed during investigation for hypertension (8). In a Chinese cohort with twenty six 17OHD patients, two patients with the complete form were normotensive (3). Varying degrees of hypertension in the 17OHD patients suggests that other factors other than the degree of P450c17 activity may be involved in the regulation of hypertension (17). Because high levels of circulating DOC saturate the mineralocorticoid receptor under most circumstances, the severity of clinical features and the age onset of hypertension and hypokalemia appear to vary, even among patients with the same mutation (5,8). Patient 1 (del exon 1-6) and Patient 5 (p.R362H) never had hypertension. It remains unclear whether this is due to the suppression of mineralocorticoid precursors by hydrocortisone, or whether it is simply because 10-15% of patients will be normotensive. 

Four of our patients reached adult height and, after sex steroid replacement, only one of these had short stature at final evaluation. The other two patients have not yet reached their final height. The short stature in our patients may be due to sex steroid deficiency. After sex steroid replacement height velocity and thus final height increased. Short stature is an unexpected finding in 17 OHD patients because sex steroid deficiency causes failure of epiphyseal fusion and bone age retardation, which is known to result in tall stature. 17OHD patients generally have normal or tall stature (6,8,18). Despite this, Ross et al (19) reported that low levels of estrogens in prepubertal Turner syndrome patients, have a positive impact on height. Sex steroids have beneficial effects on linear growth (19). While the patients in this study had normal target height SDS, 50% had short stature at presentation according to genotypic sex. All patients had delayed bone age. Although bone age retardation suggests that linear growth potential in 17OHD patients will lead to improved final height, there is no data in the literature on this issue. Interestingly Schwab et al (18) reported two German sisters, one of whom was 46,XX and the other 46,XY. At the age of ten years the 46,XY sibling had a predicted height of 203 cm. Treatment with high dose estrogens for a 13 month period resulted in a final height of 186.3 cm (18). Although tall stature may be seen in patients, especially in those with 46,XY karyotype, none of our patients had tall stature. Turkkahraman et al (9) reported three siblings with exon 1-6 deletion on the *CYP17A1* gene, the same variant found in Patient 1. One sibling had tall stature with a height SDS of 3.5 while the other two siblings had height SDS of -1.6 and -1.3 respectively (9). Patient 1 had short stature at presentation, but went on to have an improved height SDS of -1.9 on follow-up. Thus linear growth may differ even among patients with the same mutation.

We had three patients with severe mutations (exon 1-6 deletion and p.Y60Ifs*29 mutation). Height at presentation remains unknown for one of these patients, while the others had severe short stature. One patient with a point mutation (Patient 4) presented with milder short stature. Our remaining two patients had point mutations and they did not have short stature. Four of these patients had reached their adult height and two of them with the exon 1-6 deletion and one with p.Y60Ifs*29 mutation, were short while the other two patients had point mutations and normal heights. It was interesting that the heights of the patients with point mutations were less severely affected. Patient 6 was diagnosed at an early age and had a high SDS for height for age at presentation. However, the most recent examination revealed a retarded bone age and borderline short stature although this patient has not yet reached adult height. Data on long term follow up of growth of patients with 17OHD is scarce in the literature. 

Sex steroid replacement should be started at the time of puberty, in keeping with the patient’s phenotypic sex. Estrogen replacement should be followed by oral contraceptives in 46,XX patients. High levels of progesterone have negative effects on endometrium and breast tissue. Thus, some patients show breast tissue unresponsiveness despite high dose E2 treatment (20,21). In this study, breast development of the patients was at Tanner stage 1 at presentation and progressed to Tanner stage 4-5 after sex steroid replacement. Due to genetic differences, some patients had less progesterone influence on breast tissue while other tissues, such as the endometrium, were affected more extensively. This area requires further study.

In this cohort, Patient 4 reached pubic hair stage 4 at final evaluation. This patient had a DHEA-S level just below normal, which was higher than the values of the other patients all of whom had very low DHEA-S concentrations. It is reported that extreme adrenarche may develop after E2 replacement despite low androgen and DHEA-S (21). Use of dermal ointments containing estrogen have been reported to cause the growth of pubic hair in both males and females ranging from four months to two years of age (22). In light of this finding, we can speculate that estrogens also may have a stimulatory effect on hair follicles, either directly or by increasing local androgen production. In some cases adrenarche can reach the adult stage. 

Three 46,XY patients underwent gonadectomy shortly after diagnosis. These patients carry the risk of developing gonadal tumours, requiring gonadectomy at the appropriate age. Soveid and colleagues reported myelipoma of adrenal glands in addition to gonadal tumours (23). The etiology of myelipoma in CAH is still unclear, but exposure to high levels of ACTH may play a role (24). Screening of adrenals and gonadectomy at the appropriate age are important for the future health of 17OHD patients. 

In this cohort, five patients had osteoporosis at presentation. After sex steroid replacement and hydrocortisone treatment most of them improved. Cortisol and estrogen deficiency during adolescence has been associated with osteoporosis (25,26). In contrast, Wu et al (27) reported patients with 17OHD to be osteoporotic and they suggested that this may be due to the negative effect of corticosteroid replacement on bone tissue. Osteoporosis worsened with hydrocortisone and sex steroid treatment in one of our patients (Patient 6), a finding which may be due to long-term corticosteroid replacement.

We present one novel and one ultra rare gene variant with 17-OH deficiency, both described for the first time, in this study. A single base insertion in Exon 1 led to frameshift and caused premature termination of translation in Patients 2 and 3 (p.Y60Ifs*29) expected to abolish the total activity of the 17-OH enzyme. 

The missense variant (p.P409L) identified in Patient 4, affecting the Cytochrome P450 domain by altering a non-polar aliphatic amino acid proline to a non-polar amino acid leucine, resulting in an isobutyl side chain that may cause abnormal protein structure with a 3-carbon chain that loops to incorporate into the molecular backbone, is also novel. A different nucleotide alteration at the same position causing a missense change (c.1226C>G;p.P409R) has been previously reported in a few Chinese cases (15,28,29,30).

The other molecular findings, the exon 1-6 deletion in Patient 1 (9), p.R362H in Patient 5 (31) and p.G436R in Patient 6 (32) were previously reported mutations. MLPA testing is a reliable and convenient method for the diagnosis of gross deletion/duplications and is widely used when a large number of sample sets are required to be tested in a single run. qPCR is also reliable when a single patient needs to be tested for deletion and duplication and is also cost effective despite being a “boutique” test. 

Our clinical findings, together with *CYP17A1* genotypes of the patients, did not correlate with biochemical and hormone results, reflecting the expressional variation of bi-allelic mutations. Clinical and hormonal findings of two siblings (one 46,XX, the other 46,XY) with the same mutation (c.177_178insA; p.Y60Ifs*29) were also somewhat different. Findings in siblings with the same mutation revealed that the 46,XX sibling had hypokalemia and breasts at Tanner stage 3, while the 46,XY sibling was normokalemic and was at Tanner stage 4 for breast development. Moreover, the 46,XX sibling had more severe osteoporosis. Bee et al (29) reported that phenotype may vary even amongst siblings with the same mutation. 46,XY individuals have more pronounced clinical symptoms than their 46,XX siblings (29). 

Patient 1 was 46,XX and had the same mutation as three previously reported siblings (two 46,XY and one 46,XX) of Kurdish origin from Turkey. All four patients reported with this variant patients had gross partial deletion of the *CYP17A1 *gene, encompassing exons 1-6 and all of them had the same hormonal profile of complete defect of 17OHD. However, severity of the disease was different. The previously reported index patient had hypertension and hypokalemia at presentation while our patient had neither. Siblings of the index patients did not develop hypertension or hypokalemia either (9). This finding also supports the previous observations that the presence and onset of hypertension may be variable even in patients with the same genotype (8,9,29,33). In addition, exon 1-6 deletion was found in another patient of Kurdish origin from Turkey (10). The carriers of this mutation (exon 1-6 deletion) originated from Southeastern Turkey, a finding which suggests exon 1-6 deletion could be a founder mutation in Turkey.

The homozygous mutation of Patient 5 with XX chromosomal sex had the same homozygous mutation as a patient reported from Mexico (31). The Mexican case was a compound heterozygote with (R362H) and (p.K110*), presenting with severe hypertension and hypokalemia. While our patient did not have hypertension, hypokalemia was present. In our opinion it is hard to attribute hypertension to the truncating effect of the stop codon mutation. Further cases or functional analyses are needed to draw a conclusion on genotype-phenotype correlations of the mutations described thus far.

### Study Limitations

Limitations of this study include the small sample size, however it is still one of the largest pediatric cohorts, supporting to the disease has being rare.

## Conclusion

17-OHD is a rare cause of CAH and should be kept in mind in patients with pubertal delay/primary amenorrhea, hypertension and hypokalemia. Here we describe two novel mutations which both abolish *CYP17A1* protein expression in adrenal and gonadal tissue. In our experience short stature is particularly evident at presentation. Some patients may have full blown adrenarche and E2 replacement may increase the progression of adrenarche. Gonadectomy at the appropriate age is necessary in 46,XY patients who are raised as female because of the high risk of later malignancy. Due to late diagnosis, psychological problems, gender selection, hypertension and osteoporosis constitute important health problems among these patients.

## Figures and Tables

**Table 1:**
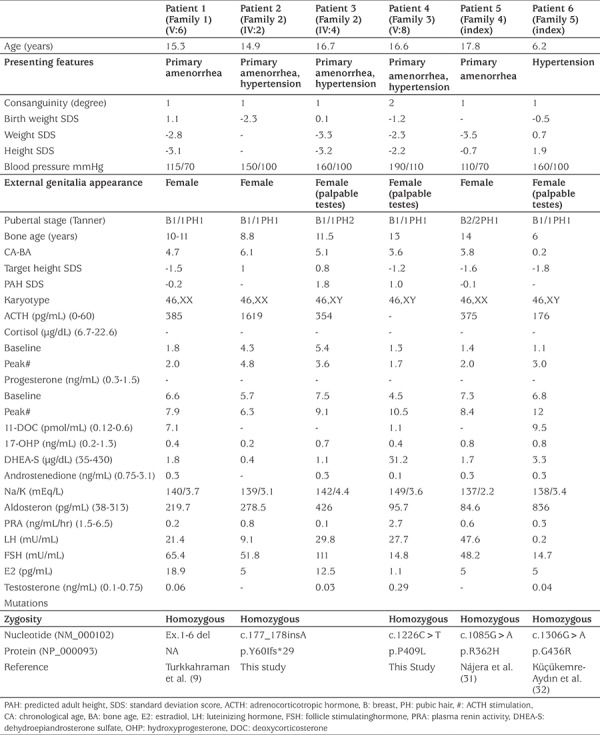
Clinical and laboratory findings of patients at presentation. Biochemical findings are given with (units) or (units) followed by (normal range)

**Table 2:**
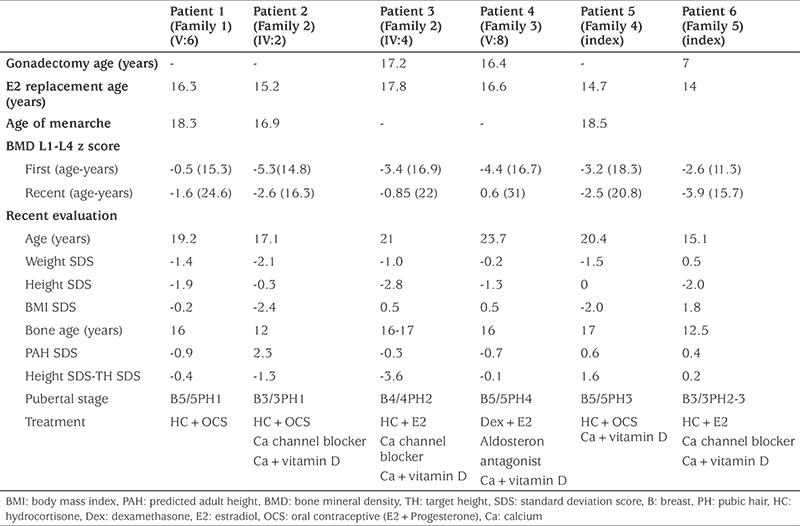
Clinical findings of the patients at follow-up
